# Protective Effects of Shen-Yuan-Dan, a Traditional Chinese Medicine, against Myocardial Ischemia/Reperfusion Injury *In Vivo* and *In Vitro*

**DOI:** 10.1155/2013/956397

**Published:** 2013-12-23

**Authors:** Hongxu Liu, Juju Shang, Fuyong Chu, Aiyong Li, Bao Wu, Xinran Xie, Weihong Liu, Hongzhi Yang, Tong Tong

**Affiliations:** ^1^Department of Cardiology, Beijing Hospital of Traditional Chinese Medicine, Capital Medical University, Beijing 100010, China; ^2^Department of Cardiology, Daxing Hospital of Traditional Chinese Medicine, Beijing 102618, China

## Abstract

*Objectives.* The study was to investigate the effects and mechanisms of Shen-Yuan-Dan (SYD) pharmacological postconditioning on myocardial ischemia/reperfusion (I/R) injury. *Methods.* In the *in vivo* experiment, myocardial injury markers and histopathology staining were examined. In the *in vitro* experiment, cell viability and cell apoptosis were, respectively, detected by 3-(4,5-dimethylthiazol-2-yl)-2,5-diphenyltetrazolium bromide (MTT) assays and Hoechst 33342 fluorochrome staining. The protein expressions of Bcl-2 and Bax were determined by immunocytochemistry assay. *Results.* Both low and high doses of SYD protected myocardium against I/R injury in rat model by reducing lactic dehydrogenase (LDH) and creatine kinase-MB (CK-MB) activity and malondialdehyde (MDA) content, increasing superoxide dismutase (SOD) activity and attenuating histopathology injury. Meanwhile, in the *in vitro* experiment, SYD promoted cell viability and inhibited the cardiomyocyte apoptosis. The level of Bcl-2 protein was restored to the normal level by SYD pharmacological postconditioning. In contrast, the Bax protein level was markedly reduced by SYD pharmacological postconditioning. These effects of SYD were inhibited by LY294002. *Conclusions.* The results of this study suggested that SYD pharmacological postconditioning has protective effects against myocardial I/R injury in both *in vivo* and *in vitro* models, which are related to activating the phosphatidylinositol 3-kinase/Akt (PI3K/Akt) pathway.

## 1. Introduction

Ischemic heart disease (IHD) is associated with high morbidity and mortality, and its prevalence is continuously increasing in China and worldwide [1]. Myocardial ischemia/reperfusion (I/R) injury is a pathophysiological phenomenon commonly seen during thrombolysis, percutaneous transluminal coronary angioplasty (PTCA), and coronary artery bypass grafting (CABG). It is defined as restoration of blood flow to a previously ischemic region followed by complex pathological events leading to tissue injury greater than the original ischemic insult [2–4]. The outcomes of the myocardium I/R injury include reperfusion arrhythmias, myocardial stunning, myocardial hibernation, and final myocardial dysfunction which inevitably result from myocardium apoptosis subsequent to I/R injury [2]. Therefore, antiapoptotic agents that prevent I/R injury may be a novel therapeutic opportunity for IHD patients.

In recent years, ischemic preconditioning (IPC) and ischemic postconditioning (IPoC) became important approaches in endogenous cardioprotection, which yield a potential to significantly reduce the I/R-induced myocardial cell damage [5–7]. Pharmacological postconditioning (PPoC) is an extension of ischemic postconditioning, in which a drug is applied to ischemic myocardium or hypoxic cardiomyocytes during the early reperfusion or reoxygenation phases, and significantly attenuates cardiomyocyte injury and apoptosis [8].

The phosphatidylinositol 3-kinase/Akt (PI3K/Akt) is a powerful survival signaling pathway in many cell types [9, 10]. Activation of the PI3K/Akt pathway may be useful to promote myocytes survival in the damaged heart [11, 12], while administration of inhibitor of PI3K-Akt pathway like wortmannin and LY294002 was reported to abolish cardioprotection caused by IPoC [13]. This highlights the potentially beneficial role of PI3K in IPoC-mediated cardioprotection [13, 14]. Inhibition of PI3K accelerates apoptosis, while activation of Akt blocks apoptosis [15].

Traditional Chinese medicine has been used in the treatment of IHD for nearly three thousands of years. Shen-Yuan-Dan (SYD), a widely used traditional Chinese medicine prescription, consists of eight crude Chinese medicinal agents named *Salvia miltiorrhiza* Bge, *Astragalus membranaceus* Bge, root of Pilose Asiabell, Radix Scrophulariae, *Hirudo nipponica* (Whitman), *Lumbricus, Eupolyphaga sinensis* (Walker), and Rhizoma Corydalis, and has been confirmed to be effective in the treatment of IHD [16, 17]. Our previous studies demonstrated that oral supplementation for four weeks with SYD decoction at 60 g per day does not only relieve symptoms of angina but also promotes recovery of cardiac dysfunction [16, 17]. This involved reduction of myocardium infarct size [18], promotion of endothelial function [19], and inhibition of oxidative injury [20]. However, the effects and mechanisms of SYD postconditioning on I/R cell apoptosis have not been clarified yet. Therefore, the purpose of our study was to examine the *in vivo* and *in vitro* effects of SYD postconditioning on protecting against myocardial I/R injury. Myocardial injury markers and histopathology staining were examined in a rat model. To further examine the involvement of the PI3K/Akt pathway in the cardioprotection by SYD postconditioning, Bcl-2 and Bax protein levels were studied by immunocytochemistry.

## 2. Materials and Methods

### 2.1. Reagents

We utilized the following reagents and assay kits: lactic dehydrogenase (LDH) detection kit (Yatai Co., Ningbo, China), creatine kinase-MB (CK-MB) detection kit (Leadmanbio Co., Beijing, China), malondialdehyde (MDA) and superoxide dismutase (SOD) detection kit (Jiancheng Co., Nanjing, China), Dulbecco's Modified Eagle Medium/Nutrient Mixture F12Ham (DMEM/F12) (Sigma Aldrich Co., St. Louis, MO, USA), fetal bovine serum (FBS) (Hyclone Co., Rockford, IL, USA), 3-(4,5-dimethylthiazol-2-yl)-2,5-diphenyltetrazolium bromide (MTT) (Amresco Co., Solon, OH, USA), hoechst 33342 (Dojindo Laboratories Co., Tokyo, Japan), rabbit anti-rat Bcl-2 IgG, rabbit anti-rat Bax IgG (Santa Cruz Co., Santa Cruz, CA, USA), Dylight 488 labeled goat anti-rabbit IgG (Goldenbridge Co., Beijing, China), and LY294002 (Biyuntian Co., Beijing, China).

### 2.2. Preparation of SYD Aqueous Extracts and Pharmacological Serum

SYD consists of eight crude medicinal agents including *Salvia miltiorrhiza* Bge (15 g), *Astragalus membranaceus* Bge (12 g), root of Pilose Asiabell (10 g), Radix Scrophulariae (5 g), *Hirudo nipponica* (Whitman) (3 g), *Lumbricus* (5 g), *Eupolyphaga sinensis* (Walker) (5 g), and Rhizoma Corydalis (5 g). All medicinal herbs were purchased from Beijing Xinglin Pharmaceutical Co. (Beijing, China) and were authenticated by Kechen Mao, a professional herbalist from Beijing TCM Hospital, Capital Medical University. The mixtures were soaked in distilled water for 30 min, boiled in 10 volumes of water (v/w) for 1 hour, and extracted three times. The filtered and mixed solution from three decoctions was concentrated under vacuum by using a rotary evaporator to a final concentration of 1 g/mL (w/v) followed by centrifugation at 3000 rpm for 30 min, which was then stored at −20°C for the following experiment.

**Figure 1:**
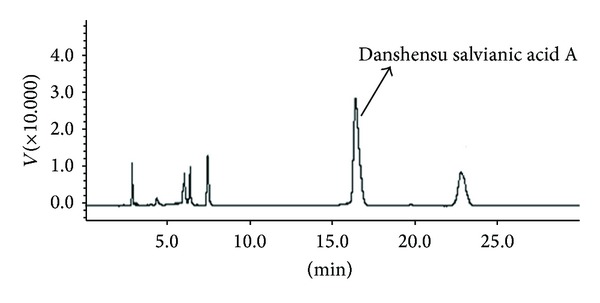
Chromatographic profile of SYD pharmacological serum. A Shimadzu LC9A series HPLC system (SHIMADZU, Japan) with a SPD-6AV detector and a Kromasil 100-5C18 column (4.6 mm × 250 mm, 5 *μ*m particle size) was used for HPLC analysis. The UV spectra were recorded in the range of 230–400 nm, and chromatographic peaks were measured at a wavelength of 280 nm. Mobile phase consisted of acetonitrile and 1% acetic acid (v/v). The flow rate was 1.0 mL/min and the column temperature was set at 25°C.

To obtain SYD pharmacological serum, fifty Wistar rats (weight: 220–250 g) which were purchased from the Institute of Laboratory of Animal Sciences, China Academy of Medical Science (Beijing, China), were divided into two groups, and SYD (6 mL/kg) or saline (6 mL/Kg) as control was administered by oral gavage, twice daily, for five days. One hour after the final administration, rats from each group were anesthetized, blood specimens were drawn from the abdominal aortic artery and centrifuged at 3000 rpm for 10 min. Serum from each rat was collected and centrifuged at 1000 rpm for another 10 min. Individual serum samples from the same treatment group were combined in a 4 mL tube, inactivated at 56°C for 30 min, and kept at −20°C until processing. Before cell experiments, both SYD and control sera were diluted to 5% or 10% (v/v) with DMEM/F12 culture medium. As there were many gradients in SYD formula, we only analyzed a main constituent in SYD pharmacological serum called Danshensu salvianic acid Afrom *Salvia miltiorrhiza* Bge by a HPLC fingerprint. Based on the fingerprint, as shown in Figure 1, we established an optimal and easily controlled procedure for preparing SYD pharmacological serum.

### 2.3. I/R Injury in Rats and Neonatal Rat Cardiomyocyte

The rat model of I/R was established according to our previously published protocol [19]. Briefly, rats were anesthetized by intraperitoneal injection of sodium pentobarbital (30 mg/kg). Then, coronary artery ligation was achieved with a gab occluder fixed onto the left anterior descending (LAD) coronary artery. A 5-0 silk suture was passed underneath the LAD (2-3 mm inferior to the left auricle) and tied. The ischemia was confirmed by myocardial blanching and ECG evidence of injury. Myocardial I/R model was induced by 30 min of ischemia followed by 3 hours of reperfusion. Rats surviving for 5 min after the reperfusion were randomized into five groups (*n* = 8 per group): sham-operated group (sham), I/R group (I/R), ischemic postconditioning (IPoC) group, SYD low-dose group (L-SYD, 3 g/kg), and SYD high-dose group (H-SYD, 6 g/kg). All drugs were administered via duodenal injection at the onset of reperfusion. Sham-operated and I/R groups were given equal volume of saline.

Primary cultures of neonatal rat cardiomyocytes from 1- to 3-days-old Wistar rats were prepared and cultured as described previously [21]. After 72 hours of cell culture, cardiomyocytes (cultured in DMEM/F12 containing 10% FBS at 37°C in CO_2_ incubation) were subjected to various treatments and subsequent experimental protocols. In order to simulate the extracellular environment of myocardial I/R injury, a simulated hypoxia/reoxygenation model was performed as described previously with some modifications [4]. Briefly, cells were randomly divided into 7 groups. Cells in the normal control group were kept in normoxic culture for 6 hours (normal). In the hypoxia/reoxygenation group (H/R), the medium was replaced with glucose-free Earle's balanced salt solution (bubbled with 95% N_2_ + 5% CO_2_ for 15 min to remove soluble oxygen) prior to hypoxia, and the cells were immediately transferred into a hypoxic incubator in a humidified atmosphere equilibrated with 95% N_2_ + 5% CO_2_ for 4 hours (hypoxia). Thereafter, Earle's solution was replaced with DMEM/F12 containing 10% FBS to simulate reperfusion, followed by normoxic culture (for reoxygenation) for 4 hours. In the next four groups, Earle's solution was replaced with DMEM/F12 + 10% FBS containing, respectively, 5% or 10% of SYD pharmacological serum (5% SYD, 10% SYD), or 5% or 10% control serum (5% control, 10% control) prior to reoxygenation with other procedures identical to the H/R group. In the LY294002 + SYD postconditioning group, Earle's solution was replaced with DMEM/F12 + 10% FBS medium containing 30 ng/mL of LY294002 and 5% SYD pharmacological serum (5% SYD + LY294002) prior to reoxygenation, while other procedures were unchanged.

Animal use conformed with the Guide for the Care and Use of Laboratory Animals (NIH Publication no. 85-23, Revised in 1996) and was approved by the Animal care and Use Committee, Beijing TCM Hospital, Capital Medical University (Beijing, China).

### 2.4. Measurement of LDH, CK-MB, SOD, and MDA

At the end of reperfusion, blood samples were drawn from the abdominal aortic artery, and serum samples were obtained by centrifugation of the specimens at 3000 rpm for 10 min at room temperature. Activities of LDH, CK-MB, SOD, and MDA were measured at 25°C using commercial kits according to the manufacturer's instructions on a spectrophotometer (Bio-Tek ELX800, Beijing, USA) at wavelengths of 340 nm (LDH and CK-MB), 532 nm (MDA), and 550 nm (SOD).

### 2.5. Histological Examination

At the end of reperfusion, rats were sacrificed. Left ventricles were sectioned, fixed for 24 hours in 10% formalin at room temperature, dehydrated by graded ethanol, and embedded in paraffin. Tissue sections (thickness of 5 *μ*m) were deparaffinised with xylene, stained with haematoxylin-eosin (H&E), and viewed under light microscopy (Leica DM2000, Wetzlar, Germany). All histological evaluations were performed in a blinded manner.

### 2.6. MTT Assay

Myocardial cells were cultured in a 96-well plate at a density of 2 × 10^5^ cells/well. Following experiments, cells were treated with 20 *μ*L MTT (5 mg/mL) and incubated for 4 hours in darkness at 37°C. Afterwards, medium and MTT were removed from the wells. The remaining MTT-formazan crystals were dissolved in 150 *μ*L DMSO (lysis for 10 min). Optical densities (OD) were analyzed spectrophotometrically at a wavelength of 540 nm, using 150 *μ*L of DMSO as blank. All experiments were performed three times.

### 2.7. Hoechst 33342 Assay

Exponentially growing cells were plated in 12-well plates at a density of 2 × 10^5^ cells/well and cultured for 72 hours. Following simulated I/R procedures, cells were fixed for 8 min with the precooled (−20°C) formaldehyde and acetone solution (1 : 1, *v/v*) and washed with PBS 3 × 3 min, followed by staining with Hoechst 33342 solution (50 mmol/L) at 37°C in darkness for 5 min. Apoptotic cells were observed and images were taken using fluorescence microscope (Olympus BX51, Tokyo, Japan), with excitation wavelength of 350 nm and emission wavelength of 460 nm.

### 2.8. Immunocytochemistry Assay

Exponentially growing cells were plated in 12-well plates at a density of 2 × 10^5^cells/well and cultured for 72 hours. Following simulated I/R procedures, cells in each group were fixed and washed with PBS as above. Afterwards, primary antibodies (rabbit anti-rat Bcl-2 or Bax IgG) were added and incubated with cells at 37°C for 60 min. Then, cells were washed three times with cold PBS, and secondary antibody (Dylight 488 labeled goat anti-rabbit IgG) was added and incubated with cells at 37°C for 20 min. Cells were observed and images were taken using fluorescence microscope. The integral optical density (IOD) was measured in fluorescence positive stained cell by Image-Pro Plus v5.0 (Media cybernetics, USA).

### 2.9. Statistical Analysis

All data are expressed as mean ± SEM. Differences between groups were analyzed by one-way ANOVA, with the Student-Newman-Keuls (SNK) assay used for post hoc analysis. The two-sided *P* < 0.05 was considered statistically significant. All analyses were performed using SPSS software (version 11.0, SPSS Inc., Chicago, USA).

## 3. Results

### 3.1. Effects of SYD Pharmacological Postconditioning on I/R Injury in Rats

**Figure 2:**
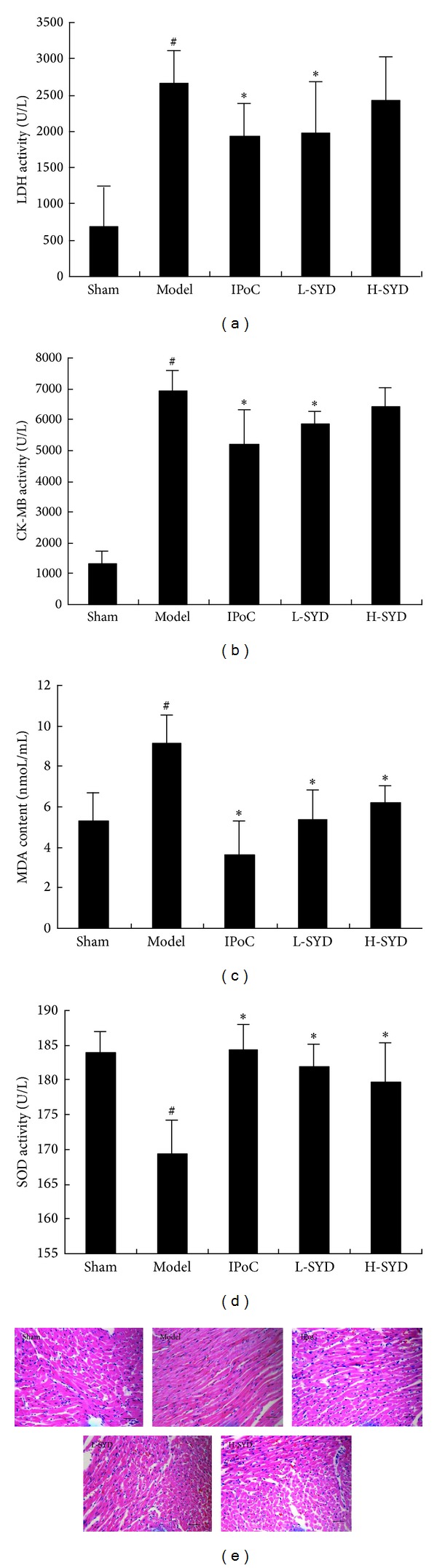
Effects of SYD postconditioning on ischemia-reperfusion (I/R) injury in the sham, I/R, IPoC, low-dose (L)-SYD (3 g/kg/day), and high-dose (H)-SYD (6 g/kg/day) groups. (a) serum LDH activity; (b) serum CK-MB activity; (c) Serum MDA content; and (d) serum SOD activity. Data are expressed as mean ± SD (*n* = 8). ^#^ *P* < 0.05 versus sham; *P < 0.05 versus I/R. (e) Representative images of H&E-stained sections. In the I/R group, myocardial fiber loss and disruption are evident, and this is reversed by SYD treatment. SYD (both L- and H-dose) was administered via duodenal injection. Scale bar represents 50 *μ*m.

As a consequence of I/R injury, serum activities of LDH and CK-MB were significantly increased in I/R group as compared with sham group (*P* < 0.05; Figures 2(a) and 2(b)). Both IPoC and low dose of SYD (3 g/kg) significantly inhibited elevation of LDH and CK-MB activity (*P* < 0.05 versus I/R group; Figures 2(a) and 2(b)), while no significant difference was found between H-SYD group and I/R group with regard to these markers (Figures 2(a) and 2(b)). After I/R, MDA activity increased, activity of SOD decreased, in the I/R group compared with sham group (*P* < 0.05; Figures 2(c) and 2(d)), while both IPoC and SYD treatments (L- and H-dose) significantly inhibited elevation of MDA activity and promoted SOD activity compared with the I/R group (*P* < 0.05; Figures 2(c) and 2(d)).

Three hours after reperfusion, no lesions were observed in the sham group. In the I/R group, apparent perivascular edema and structural disarray were present, and neutrophil influx was documented. After treatment with SYD (both L- and H-dose), histological features became typical of normal cardiac structure or mild architectural damage (Figure 2(e)).

### 3.2. Effects of SYD Pharmacological Serum on Cell Viability in H/R Cardiomyocytes

**Figure 3:**
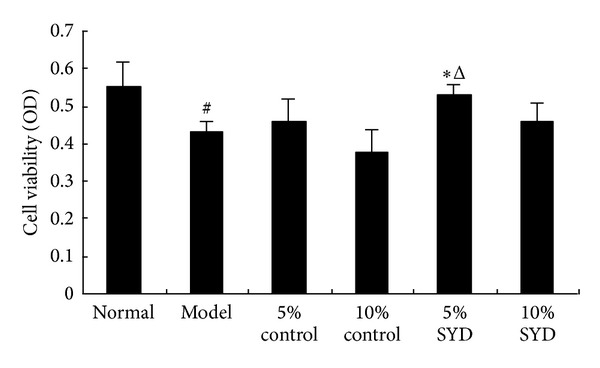
Effects of different doses of SYD pharmacological serum on cell viability in H/R cardiomyocytes. The cells were exposed to 6 hours of normoxic culture (normal), 2 hours of hypoxia followed by 4 hours of reoxygenation (H/R), and postconditioning with two concentrations of SYD or control serum (5% or 10% each). Data are expressed as mean ± SEM (*n* = 3). ^#^ *P* < 0.05 versus normal, *P < 0.05 versus 5% control serum, and ^Δ^ *P* < 0.05 versus 10% SYD.

Effects of SYD pharmacological postconditioning on cell viability in H/R myocardial cells were assessed by MTT assay. As shown in Figure 3, viability was decreased in H/R group compared with normal group (*P* < 0.05), and 5% SYD postconditioning significantly increased cell viability compared with 5% control or 10% SYD groups (*P* < 0.05, in both comparisons). The results indicated that SYD preserves cell viability effect, however, only at an optimal dose.

### 3.3. Effects of SYD Pharmacological Serum and LY294002 Postconditioning on Apoptosis in H/R Cardiomyocytes

**Figure 4:**
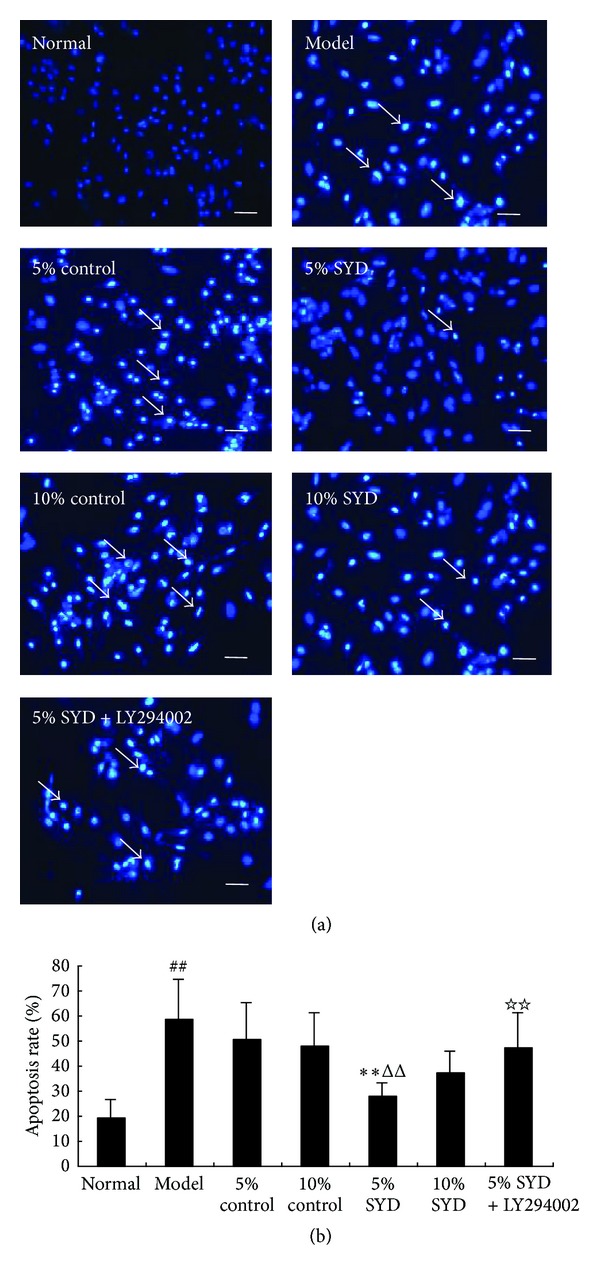
Effects of SYD and LY294002 on cell apoptosis. (a) Representative images of Hoechst 33342 staining. (b) Quantitative analysis of apoptosis rate. The cells were exposed to 6 hours of normoxic culture (normal), 2 hours of hypoxia followed by 4 hours of reoxygenation (H/R), postconditioning with two concentrations of SYD or control serum (5% or 10% each). In some experiments, 5% SYD was complemented with LY294002 (5% SYD + LY294002). Data are expressed as mean ± SEM (*n* = 3), ^##^ *P* < 0.01 versus normal, **P < 0.01 versus 5% control serum. ^ΔΔ^ *P* < 0.01 versus 10% SYD, and ^☆☆^ *P* < 0.01 versus 5% SYD. Scale bar represents 50 *μ*m.

Effects of SYD postconditioning on the H/R induced cell apoptosis were assayed by Hoechst 33342 staining. As shown in Figure 4, few apoptotic cells were present in the normal group. As expected, there were many apoptotic (i.e., Hoechst 33342-positive) cells in the H/R group and in the group that received 5% SYD + LY294002 postconditioning. By contrast, fewer apoptotic cells were observed when SYD alone was used for postconditioning (*P* < 0.01; Figure 4(a)). Further, 5% SYD exhibited a better effect compared with postconditioning with 10% SYD group (*P* < 0.01; Figure 4(b)).

### 3.4. Effects of SYD Pharmacological Postconditioning on Expressions of Bcl-2 and Bax in H/R Cardiomyocytes

**Figure 5:**
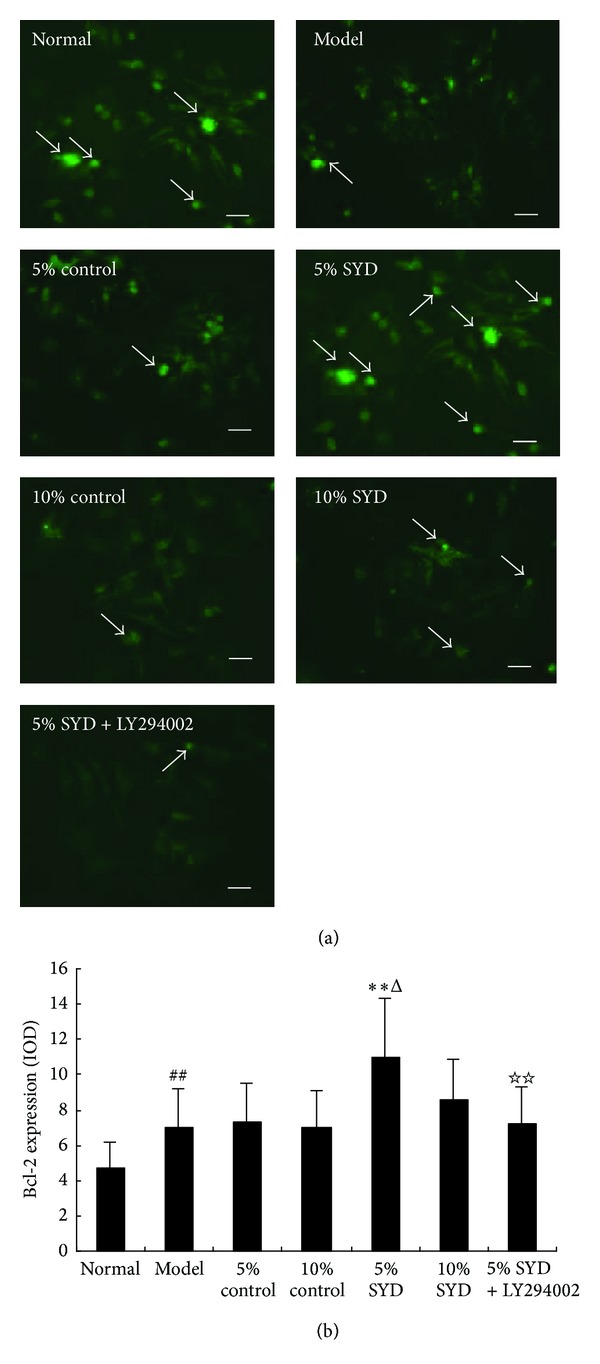
Effects of SYD and LY294002 on Bcl-2 expression. (a) Representative images of Bcl-2 staining. (b) Quantitative analysis of Bcl-2 expression (IOD). The cells were exposed to 6 hours of normoxic culture (normal), 2 hours of hypoxia followed by 4 hours of reoxygenation (H/R), and postconditioning with two concentrations of SYD or control serum (5% or 10% each). In some experiments, 5% SYD was complemented with LY294002 (5% SYD + LY294002). Data are expressed as mean ± SEM (*n* = 3). ^##^ *P* < 0.01 versus normal, **P < 0.01 versus 5% control serum, and ^ΔΔ^ *P* < 0.01 versus 10% SYD, ^☆☆^ *P* < 0.01 versus 5% SYD. Scale bar represents 50 *μ*m.

**Figure 6:**
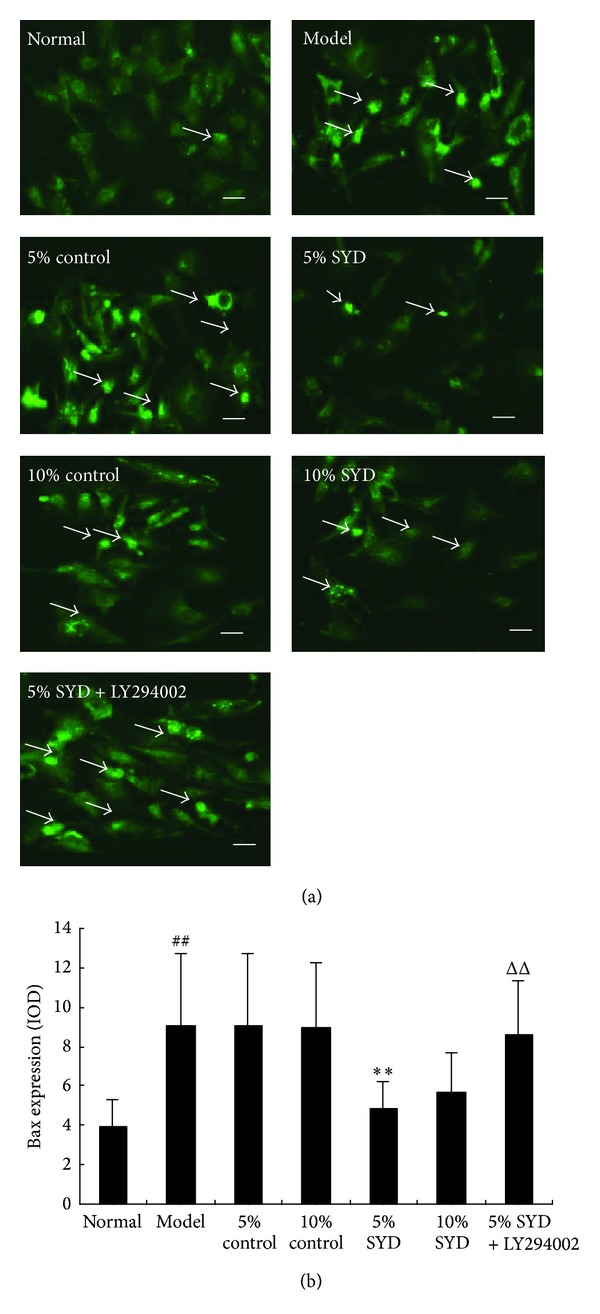
Effects of SYD and LY294002 on Bax expression. (a) Representative images of Bax staining. (b) Quantitative analysis of Bax expression (IOD). The cells were exposed to 6 hours of normoxic culture (normal), 2 hours of hypoxia followed by 4 hours of reoxygenation (H/R), and postconditioning with two concentrations of SYD or control serum (5% or 10% each). In some experiments, 5% SYD was complemented with LY294002 (5% SYD + LY294002). Data are expressed as mean ± SEM (*n* = 3). ^##^ *P* < 0.01 versus normal, **P < 0.01 versus 5% control serum, ^ΔΔ^ *P* < 0.01 versus 10% SYD, and ^☆☆^ *P* < 0.01 versus 5% SYD. Scale bar represents 50 *μ*m.

The Bcl-2 and Bax positive cells were observed in cytoplasm of cells cultured under normoxic conditions (Figures 5(a) and 6(a)). In the SYD postconditioning group, expression of Bcl-2 was significantly increased compared with H/R group (*P* < 0.01; Figure 5(a)), while expression of Bax was significantly decreased (*P* < 0.01; Figure 6(a)). By contrast, expression of Bcl-2 was decreased when pre-conditioning with 5% SYD complemented with LY294002, whereas expression of Bax was increased (*P* < 0.01; Figures 5 and 6). The pattern of the changes in Bcl-2 and Bax expressions was compatible with the patterns observed in the cell viability and apoptosis experiments.

## 4. Discussion

Myocardial I/R injury can be defined as damage to the heart when blood supply is restored after a prolonged period of ischemia resulting in oxidative damage, inflammation, cell apoptosis, and cardiac dysfunction [3, 22]. IPoC is defined as brief episodes of coronary occlusion and reperfusion at the onset of reperfusion after sustained ischemic insult and has been confirmed to have beneficial effects in protecting against I/R injury in dogs, cats, rats, and rabbits [6, 23–25]. Further, PPoC is a condition when a drug is applied to ischemic myocardium or hypoxic cardiomyocytes during the early reperfusion or reoxygenation phases; PPoC has similar protective effects in attenuating cardiomyocyte injury and apoptosis. And a diverse array of pharmacological agents such as bradykinin-B2 receptor activator [26], PKC-adenosine A2b receptor activator [27], phytoestrogen genistein [28], and several natural drug components administered at the time of reperfusion was reported to be cardioprotective [29–31]. Our previous studies confirmed that SYD, an adjunctive traditional Chinese medicine prescription in the treatment of ischemic heart disease, has beneficial effects in reducing myocardium infarct size [18], promoting endothelial function [19], and inhibiting oxidative damage [20]. The mechanisms behind these beneficial effects include activation of the PKC signaling [18].

Traditional Chinese medicine (TCM) has been widely used in treating many kinds of cardiovascular and metabolic diseases, such as hypertension, hyperlipidemia, and diabetes [32–34]. Shen-Yuan-Dan (SYD), a widely used traditional Chinese medicine prescription, has been confirmed to be effective in management of IHD, but the effects of SYD postconditioning on myocardial I/R injury and cell apoptosis and the involved mechanism still remain unclear. In the present study, we investigated the effects and mechanisms of SYD postconditioning in protecting from myocardium I/R injury and apoptosis, both *in vivo* and *in vitro*. Our findings demonstrate that both low (3 g/kg) and high dose (6 g/kg) of SYD protected myocardium against I/R injury in rat model, as demonstrated by reduced serum LDH and CK-MB activity and MDA content, increased SOD activity and attenuated histopathology injury. In the *in vitro* studies, SYD pharmacological serum promoted cell viability and inhibited the cardiomyocyte apoptosis. Expression of Bcl-2 in cells treated with SYD pharmacological serum was significantly increased, while Bax expression was markedly reduced. These effects of SYD were inhibited by LY294002, an inhibitor of PI3K/Akt. The above results suggest that SYD is capable of protecting myocardium from I/R injury and inhibit the H/R-induced cell apoptosis and that this protection involves activating PI3K/Akt signaling pathway.

LDH and CK-MB are two specific myocardium injury biomarkers, which are often elevated in myocardial infarction and other ischemic injuries [35]. It is reported that myocardial I/R injury can also significantly increase the activity of LDL and CK-MB, and evidence from previous studies confirmed that IPC and IPoC can significantly reduce the activity of LDH and CK-MB and attenuate histopathology injury in myocardium, indicating great potential of IPC and IPoC in the treatment of I/R injury [36]. As described above, both low and high doses of SYD significantly inhibited the elevation of LDH and CK-MB in our study.

It is widely accepted that oxidative stress, which is associated with increased formation of reactive oxygen species (ROS), plays an important role in the pathogenesis of I/R injury [37]. Various lines of clinical and experimental evidence suggested that myocardium injury after I/R can be attributed to oxygen-free radicals mediated lipid peroxidation, a process that can be measured through its by-products, specifically MDA, and the activity of endogenous antioxidant enzymes such as SOD can be decreased after I/R injury [37]. In our study, the MDA content was significantly increased and SOD activity was significantly decreased in I/R group and both low and high dose of SYD can significantly decrease the serum MDA content and increase SOD activity at the end of the experiment. The results indicated that SYD postconditioning is protective in attenuating the reperfusion mediated lipid peroxidation damage.

Apoptosis has been shown to play an important role in the pathogenesis of myocardial I/R injury [38]. The balance between the up- and downregulations of the members of proapoptotic (Bax and Bad) and antiapoptotic (Bcl-xL and Bcl-2) family proteins determines the fate of the cells either to undergo apoptosis or to survive [38]. It is reported that the process of cell apoptosis after myocardial I/R showed a remarkable decrease of Bcl-2 gene expression and increased expression of Bax, and evidence from various *in vivo* and *in vitro* studies have confirmed the beneficial effects of ischemic postconditioning and pharmacological postconditioning in protecting against I/R injury mediated apoptosis [39]. In this study, we first investigated the effects of different concentrations of SYD pharmacological serum in protecting against simulated hypoxia/reoxygenation injury *in vitro* by MTT assay. The results showed that simulated hypoxia/reoxygenation (I/R *in vitro*) can significantly decrease the cell viability in cultured cardiomyocyte and postconditioning with different concentrations (5% and 10%) of SYD pharmacological serum greatly decreased the loss of cell viability (Figure 3). These results indicate that SYD pharmacological postconditioning significantly protected cardiomyocyte from I/R-induced cytotoxicity.

PI3K/Akt plays a key role in the reperfusion injury salvage kinase (RISK) pathway. Its activation leads to cardiac protection during myocardial I/R injury [8]. Our experiments confirmed that protective effects of SYD pharmacological serum were associated with PI3-kinase/Akt signaling pathway, as its inhibition reversed beneficial effects of SYD.

Finally, our study also demonstrated some interesting results. Comparing with postconditioning with 10% SYD, 5% SYD better improved the rate of myocardial cells' survival and more efficiently reduced the rate of apoptosis.

In conclusion, the results of our study demonstrate that SYD has a beneficial effect in protecting ischemic myocardium from the I/R injury and inhibiting cell apoptosis in H/R cardiomyocytes. The mechanism by which SYD exhibits its cardioprotective effects is associated with activation of the PI3K/Akt pathway. Better effects were observed in the low-dose SYD indicating that an optimal treatment dose may be needed for maximal cardioprotection.
